# Developing standards for reporting implementation studies of complex interventions (StaRI): a systematic review and e-Delphi

**DOI:** 10.1186/s13012-015-0235-z

**Published:** 2015-03-30

**Authors:** Hilary Pinnock, Eleni Epiphaniou, Aziz Sheikh, Chris Griffiths, Sandra Eldridge, Peter Craig, Stephanie JC Taylor

**Affiliations:** Asthma UK Centre for Applied Research, Allergy and Respiratory Research Group, Centre for Population Health Sciences, University of Edinburgh, Doorway 3, Medical School, Teviot Place, Edinburgh, EH8 9AG Scotland UK; Centre for Primary Care and Public Health, Blizard Institute, Barts and The London School of Medicine and Dentistry, London, UK; Pragmatic Clinical Trials Unit, Centre for Primary Care and Public Health, Blizard Institute, Barts and The London School of Medicine and Dentistry, London, UK; MRC/CSO Social and Public Health Sciences Unit, University of Glasgow, Glasgow, UK

**Keywords:** Reporting standards, Implementation science, Dissemination and implementation, Complex interventions

## Abstract

**Background:**

Dissemination and implementation of health care interventions are currently hampered by the variable quality of reporting of implementation research. Reporting of other study types has been improved by the introduction of reporting standards (e.g. CONSORT). We are therefore developing guidelines for reporting implementation studies (StaRI).

**Methods:**

Using established methodology for developing health research reporting guidelines, we systematically reviewed the literature to generate items for a checklist of reporting standards. We then recruited an international, multidisciplinary panel for an e-Delphi consensus-building exercise which comprised an initial open round to revise/suggest a list of potential items for scoring in the subsequent two scoring rounds (scale 1 to 9). Consensus was defined *a priori* as 80% agreement with the priority scores of 7, 8, or 9.

**Results:**

We identified eight papers from the literature review from which we derived 36 potential items. We recruited 23 experts to the e-Delphi panel. Open round comments resulted in revisions, and 47 items went forward to the scoring rounds. Thirty-five items achieved consensus: 19 achieved 100% agreement. Prioritised items addressed the need to: provide an evidence-based justification for implementation; describe the setting, professional/service requirements, eligible population and intervention in detail; measure process and clinical outcomes at population level (using routine data); report impact on health care resources; describe local adaptations to the implementation strategy and describe barriers/facilitators.

Over-arching themes from the free-text comments included balancing the need for detailed descriptions of interventions with publishing constraints, addressing the dual aims of reporting on the process of implementation and effectiveness of the intervention and monitoring fidelity to an intervention whilst encouraging adaptation to suit diverse local contexts.

**Conclusions:**

We have identified priority items for reporting implementation studies and key issues for further discussion. An international, multidisciplinary workshop, where participants will debate the issues raised, clarify specific items and develop StaRI standards that fit within the suite of EQUATOR reporting guidelines, is planned.

**Registration:**

The protocol is registered with Equator: http://www.equator-network.org/library/reporting-guidelines-under-development/#17.

## Background

The UK Medical Research Council (MRC) provides guidance to help funders, researchers and policymakers make appropriate decisions in relation to developing, evaluating and implementing complex interventions [1]. Randomised controlled trials (RCTs), the gold standard of research designs for assessing the efficacy/effectiveness of interventions [2], are typically delivered under tightly controlled conditions, with carefully selected, highly motivated, fully informed and consented participants, and typically follow detailed and relatively rigid protocols to avoid the influence of confounding variables and limit the impact of bias [3,4]. Implementation studies that accommodate - or even encourage - diversity of patient, professional and health care contexts in order to inform implementation in real-life settings are relatively uncommon [5]. Using a range of methodologies [1], implementation interventions are delivered within the context of routine clinical care and accessible to all patients clinically eligible for the service (as opposed to participants selectively recruited into a research study). By comparing a new service/procedure with the existing/previous regime, and assessing process, clinical and population level outcomes [6,7], they provide practical information about the impact on time and resources, the training requirements and workplace implications of implementing interventions into routine care [4,8]. They are useful study designs when developing policy recommendations [7].

The standard of reporting of implementation studies has been criticised as being incomplete and imprecise [9-11]. Specific issues include inconsistent use of terminology making it difficult to identify sensitive and specific terms for search strategies [5], a lack of clarity about the methodology making it difficult to determine if a study was testing the implementation of an initiative, and a poor description of the intervention being implemented so that replication would not be possible [12]. Introduction of the consolidated standards of reporting trials (CONSORT) checklist [13,14] has standardised the reporting of trials, with some evidence that standards have improved [15-19]. The success of this initiative has encouraged the development of further reporting standards, but although some can inform aspects of implementation science (e.g. observational studies [19], quality improvement studies [20] and non-randomised public health interventions [21]), none adequately address the reporting of implementation studies [11]. To address this gap, we are developing the standards for reporting implementation studies (StaRI) statement [22], the first phases of which were a literature review and e-Delphi exercise.

## Methods

We followed the methodology described in the guidance for developing health research reporting guidelines [23]. A more detailed description of our methods is available on the EQUATOR website [22].

### Literature review in order to identify potential standards

We undertook a literature review to identify evaluations of the standard of implementation study reporting and expert opinion on current design and reporting practice. We searched the MEDLINE database, using guideline terms such as ‘standard*’, ‘*guidance*’; ‘*framework*’; ‘reporting guideline*’; (report* ADJ GOOD ADJ PRACTICE) AND study design such as ‘*implementation*’; ‘*implementation science*’; ‘*Phase IV*’; ‘*Phase 4*’; ‘*real-life*’; ‘*routine clinical care*’; (‘*real-world*’ or ‘*real world*’ or *routine* or *nationwide*) *adj1* (setting* or *practice* or *context*). We explored existing EQUATOR statements [13,14,19-21] and undertook snowball searches from their reference lists and, in addition, hand searched the *Journal of Implementation Science*, *Pragmatic and Observational Research*, *Quality and Safety in Healthcare.*

We identified potential standards from the literature review and collated them as possible standards for inclusion in a StaRI Statement.

### International expert panel

We recruited, by e-mail, an international expert panel to include professionals involved with the design and evaluation of complex interventions [1], journal editors from high impact general and implementation specific journals, researchers who have published high-profile implementation research, representatives of funding bodies, guideline developers and authors of related EQUATOR standards [13].

### e-Delphi exercise to identify and prioritise standards

Originating from the RAND Corporation in the 1950s [24], the Delphi method is a technique in which an expert panel contributes ideas and then ranks suggestions in successive rounds until pre-defined consensus is reached [25-27]. The panellists work independently, and their contributions are anonymous, but in each round, responses are influenced by summary feedback from previous rounds. We used Clinvivo systems [www.clinvivo.com] to facilitate the web-based process, which (following piloting by the study team to ensure optimal terminology and clarity) involved an open round and two scoring rounds. Participants were encouraged to complete all rounds of the exercise.

#### Open round

The first round invited the expert panel to contribute potential standards which should be required in reporting implementation studies. To aid deliberation, we provided the provisional standards derived from the literature review, collated under appropriate headings (e.g. rationale and underpinning evidence for the study, description of setting, recruitment, intervention, outcomes and data collection, presentation of results and interpretation). Ample free text space was provided to enable participants to provide their own suggestions and to comment on the exemplars.

Responses were collated by HP, reviewed by the research team, and a checklist of potential items derived for ranking in the first scoring round.

#### First scoring round

Panel members were asked to score each item on the checklist on a scale of 1 to 9 (i.e. least important to very important). There was an opportunity at the end of each section of the checklist to add any comments or suggest any further standards that the respondent felt should be considered. Reminders were sent a few days before and immediately after the 2-week deadline. The median score and a graphical display of the distribution of responses were prepared for feedback in the next round.

#### Second scoring round

Participants who completed the first scoring round were sent a second round checklist in which the median result from the first scoring round was listed alongside the participant’s own score for each item. Participants were invited to reconsider the importance of the standards and confirm or revise their score in the light of the group opinions. Reminders were sent a few days before and immediately after the 2-week deadline.

We anticipated that two scoring rounds would allow an acceptable degree of agreement on priority items, but if not, a final third scoring round was planned. This would follow the format of the second scoring round, but omit items that had 80% agreement with the low priority scores of 1, 2, or 3.

#### Quantitative analysis of scoring

Participants were advised that scores of 7 to 9 were defined as indicating that they had ‘prioritised’ that item, and conversely, scores of 1 to 3 were defined as ‘rejection’ of an item. We calculated the proportion of respondents prioritising each item: consensus was defined as 80% agreement for the priority score of 7 or more.

#### Qualitative analysis of free-text comments

The free text comments from the open round and the two scoring rounds were coded (by HP and reviewed by the research team) and thematically analysed to identify the key issues from the perspective of the participants.

## Results

### Literature review initial list of potential standards

**Figure 1:**
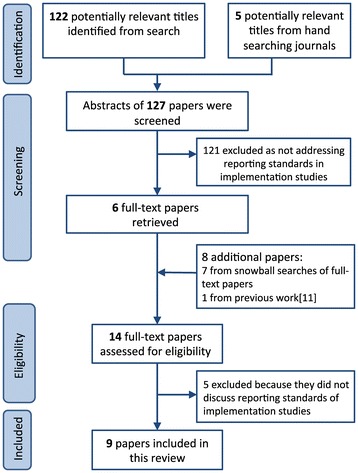
**PRISMA flow chart.**

See Figure 1 for the PRISMA flow chart. We screened the titles and abstracts of 127 papers and included six for full-text screening. Snowball searches from these six identified a further seven papers. Five of these 13 papers were excluded because on reading the full text, they did not discuss standards of reporting implementation studies. We thus included nine papers: four were discussion papers [4,11,28,29], two were editorials [7,10], two were methodological papers [9,30] and one was an online source [31]. The common theme was the importance of improving the standard of reporting in implementation research.

Table 1 summarises the standards identified from the literature review. We collated these to define a list of 36 suggested items which were included as exemplars in the initial e-Delphi process.

**Table 1:** **Summary of checklist items suggested by the literature**

**Checklist section**	**Suggested checklist item**
Introduction	
Background	Report the care or quality gap (e.g. failure to implement guidelines) that the intervention seeks to address [29].
	Report the evidence behind the intervention to be implemented (e.g.: RCTs, controlled trials, systematic reviews) [11,29].
	Report the theory behind the intervention [10,31,29] and how the theory influenced the study design [10].
Methods	
Setting	Describe the study setting [10,11,31].
	Describe any changes or modifications required to adjust to the intervention [7,10].
	Report readiness to change (also those not eager to change) [29].
	How did the setting enable or hinder the implementation? [10]
Participants	Report method of recruitment [4] which should be to the clinical service not the research [11]. Participants recruited to research should be considered as a sub-group [11].
Intervention	Provide detailed description of intervention strategies:
	Components/content for each group [10,31,29].
	Frequency [10], duration [4,10,31,29] and intensity [10,31,29] of the intervention
	Mode of delivery [31,29] and materials used [31,29].
	What is the relationship of components to the theory discussed above? [10]
	What is the target population? [4,11]
	Provide details of participants receiving the intervention (eligibility criteria?) [10,11,31].
	Provide detailed characteristics of those promoting and undertaking the intervention [4,10,31].
	What were the processes for ensuring intervention fidelity? [4,7,10,31]
	Describe the intervention received by control group (not just describe as ‘usual care’) [31].
	The intervention must be delivered as part of routine care [11].
Analysis	Describe measurements used to assess implementation effectiveness [29].
	Outcomes should be assessed at the population level, typically with routine data [11]. Describe methods of statistical analysis [31,29].
Results	
Participant flow	Report the process by which eligible patients were invited to participate (e.g. number invited and timescale) and any exclusions [4].
	Report participation rate among the eligible population [4,11] and the representativeness of participants compared to whole eligible population [4].
Setting	Report characteristics of the setting to enable assessment of representativeness [4].
Intervention	Cost of the intervention [4].
	Report any modifications or adaptation [4,11,28].
	Report any intervention feasibility, acceptability, and potential sustainability [28].
Discussion	
Reflections	Reflect on the implementation process [10,11].
	Report any lessons learned from the implementation process [10].
	Report any barriers or facilitators for implementing in routine care [9].
	Discuss the relationship between adaptations/modifications and treatment outcomes, implementation outcomes and adverse events [28].

### International expert panel

Of the 66 international experts approached, 23 agreed to participate and contributed to the open round. Some were invited by virtue of their position in an organisation, and we accepted their suggested deputies if they personally were unable to help. The resultant panel was international (United Kingdom (*n* = 12), United States (*n* = 7), Australia and New Zealand (*n* = 2), Netherlands (*n* = 2)) and multidisciplinary (health care researchers (*n* = 19), journal editors (*n* = 7), health care professionals (*n* = 5) methodologists (*n* = 6), guideline developers (*n* = 3), charity funders (*n* = 3), health care managers (*n* = 2) and national funding bodies (*n* = 2): many participants contributed more than one perspective).

Twenty respondents (87%) completed the first scoring round and 19 (83%) completed the second scoring round.

### e-Delphi exercise to identify and prioritise standards

#### Open round

All the 36 items suggested by the literature review attracted comment (both agreement and disagreement), and additional suggestions were made. As a result of these comments, four of the original items were rejected, 23 were revised and 15 additional items were included. A total of 47 potential items thus went forward to the scoring rounds.

#### Consensus (scoring rounds)

Table 2 lists the 35 items that achieved the *a priori* level of consensus for inclusion, i.e. 80% agreement with scores 7, 8 or 9: 19 items achieved 100% agreement. No items were rejected by 80% of the respondents: most of the others (see Table 3) scored in the equivocal range of 4, 5 or 6.

**Table 2:** **The 35 items which achieved consensus**

**Section**	**Standard**	**Consensus (% agreement with scores 7, 8 or 9)**
Title and abstract	There should be a structured abstract which clearly states aim, study design, setting, population, intervention, outcomes, conclusion and implications.	95%
Introduction	Identify the aspect of care that the new service being implemented aims to address (e.g. implementing a guideline recommendation or evidence-based management).	90%
	Critically report the evidence underpinning the new service to be implemented: (e.g. phase III randomised controlled trials, systematic review, guideline recommendations).	100%
	Describe the rationale for the new service design.	95%
	Report the implementation strategy used and its underpinning theory.	84%
	Clearly define the aims of the study, differentiating between implementation (process) objectives and effectiveness (clinical) objectives.	100%
Method (setting)	Describe the study setting (including health service, personnel involved, patient and public involvement, demography of patients, etc.).	100%
	Give year(s) during which the new service was implemented (i.e. planned, initiated and actively developed) and followed up.	95%
Methods (the new service)	Describe the new service (e.g. components/content, frequency, duration, intensity, mode of delivery, materials used) with advice on accessing additional detailed information. Use of a standardised checklist (e.g. TIDieR) is recommended.	100%
	Describe the professional backgrounds, roles and training requirements of the personnel involved in delivering the intervention with advice on accessing additional detailed information.	84%
	Define the core components of the intervention, and the processes for assessing fidelity to this core content, and what, if any, local adaptation was allowed.	100%
	Describe the intervention received by control/comparator group not simply stating ‘usual care’.	95%
Methods (population)	Describe sites invited/excluded with reasons.	100%
	Describe the population targeted by the intervention and any eligibility criteria.	100%
	Report method by which patients are referred to or access the new service.	100%
Methods (randomisation)	Description of randomisation (or if not randomised how comparator group was selected).	95%
Methods (data)	Describe outcome measurements (specifically describing any that are at population level) distinguishing between process and clinical outcomes and health economic data.	100%
	Describe data collection processes (specifically including methods of extracting routine data).	100%
	Describe any processes for quality assurance (especially for use of routine data).	84%
Methods (analysis)	Describe power calculation and rationale for sample size.	100%
	Describe methods of statistical analysis (with reasons for that choice including approach to clustering, handling of missing data, intention to treat analysis, and adjustment for confounders, etc.).	100%
	Specify *a priori* sub-group analyses (e.g. between different sites in a multicentre study, different clinical or demographic populations).	95%
Results (population)	Report the number of sites approached, reasons for non-participation and characteristics of participating sites.	89%
	Report the total eligible population (e.g. number of people with the relevant condition registered with the practice, or eligible for a service), number approached and any exclusions.	100%
	Report participation rate among the eligible population, compare characteristics with the eligible population as a whole and describe any known reasons for non-participation.	95%
	Report compliance with/attrition from the service as a process outcome.	95%
	Include a CONSORT diagram (modified as necessary) to illustrate the recruitment of sites, provision of service to patients and any sub-groups.	84%
Results (fidelity)	Report fidelity to the core components of the planned intervention (including, in multicentre studies, in the different settings).	100%
	Report any modifications or adaptations to the new service during the course of the study.	100%
Results (outcomes)	Report outcomes for the whole eligible population before an analysis of any sub-groups.	100%
	Report process and clinical outcomes.	100%
	If relevant, report impact on use of health service resources (and ideally cost of the intervention).	84%
	Report any unintended consequences or adverse effects.	100%
Discussion	Interpret findings in the light of the general body of literature and consider implications for health care services (including issues of generalizability, transferability, strategies for facilitating and normalising into routine care).	100%
General	Include statement(s) on regulatory approvals (including, as appropriate, ethical approval, confidential use of routine data, governance approval), trial/study registration, funding and conflicts of interest.	89%

**Table 3:** **Items which did not achieve consensus**

**Section**	**Standard**	**Consensus (% agreement with scores 7, 8 or 9)**
Title and abstract	The title (or abstract if word count of title precludes) should include a description of the methodology (e.g. phase IV implementation study, cluster randomised implementation trial, interrupted time series, before and after, stepped wedge study).	79%
Introduction	Include a description of the wider health care/policy/commercial context.	58%
	Describe any pilot implementation work and the conclusions from that work.	63%
Methods (the new service)	What is the relation of components of the intervention to the rationale for the new service design and/or theory underpinning implementation discussed above?	30%
	Define role of the researchers in design and implementation.	79%
Methods (population)	If applicable, describe any consent required (which should be to the new service and not to research).	53%
	Describe recruitment of any sub-groups recruited for additional research tasks (e.g. questionnaire completion, physiological measures, detailed record analysis).	47%
Results (population)	Report details of any subgroups recruited to specific research tasks (e.g. questionnaire completion, physiological testing) as opposed to the clinical service. Compare characteristics of any sub-groups to the whole eligible population.	74%
Discussion (population)	Include a structured abstract (for example including summary of findings, strengths and limitations, comparison with other studies, conclusions and implications).	58%
	Reflect on the processes of implementing the service, barriers or facilitators and lessons learned.	79%
	How did the setting enable or hinder the implementation of the new service?	79%
	How was the new service was implemented highlighting (if relevant) variations between sites and over time and the impact on treatment outcomes and unintended consequences?	74%

### Over-arching issues raised by the expert panel

In addition to specific comments related to individual items, thematic analysis of the free text comments revealed a number of over-arching issues

#### Dual aims of implementation and effectiveness

A distinction was made between the assessment of implementation (measured by process outcomes) and assessment of effectiveness (measured by clinical outcomes) with most comments supporting the concept that both constructs were important in implementation research. Cost-effectiveness was specifically highlighted as essential information for health services.

‘*This should clarify the “effectiveness aims” from the “implementation aims.”*’ [Open round comment]

‘*Analyses must examine impacts/outcomes as well as processes*’ [Open round comment]

‘[Cost-effectiveness] *is important for (governmental) health agencies, funding agencies, insurance companies, but also for healthcare centres and health researchers themselves*’ [Open round comment]

#### Balancing the need between detailed descriptions and the risk of overload

Nine of the 35 prioritised items focussed on the requirement for a description of the novel features of the intervention, including details of the setting, the target population, stakeholder engagement and service delivery. These details were described by the expert panel as ‘useful’, and comprehensive descriptions were considered to be ‘ideal’ as they enabled ‘a better judgement to be made about the added value of the new service’, however, it was widely recognised that space restrictions in a journal article might make detailed descriptions impractical especially for ‘large-scale interventions’. A number of alternative strategies were suggested including a ‘brief description in the methodology section’ and providing details in a separate paper, an appendix or ‘web extra’ or ‘available from the authors on request’.

‘*Sorry, lots of essentials in my response. Hard to say much shouldn't be there really, good suggestions for a reporting standard*’ [First scoring round comment]

‘*Only concerns - all individual items are fine - is the overall cumulative burden compared to journal space usually available*’ [Open round comment]

#### Fidelity to the intervention vs. adaptation to the new service

The item related to fidelity and the item reflecting modification and/or adaptation of the intervention both achieved 100% consensus as priority items, though they both generated a range of comments. In general, fidelity and adaptation were seen as separate, equally important constructs, though at least one respondent considered that they could be combined as they both reflected whether the intervention was ‘delivered as intended’. Some comments linked fidelity with ‘standardisation’ and suggested that variation was ‘a failure to adhere to intended service model’. Others emphasised the inevitability (or even desirability) that an intervention would be adapted by different settings and advocated using ‘non-judgemental’ terminology to describe the diversity of implementation. Time was identified by several respondents as an additional dimension. It was also highlighted that modifications could be ‘unintended’ as well as planned variation between centres.

‘*Also the concept of fidelity and implicit demand to not report variations - which we know happen everywhere*’*.* [Open round comment]

‘*It is inevitable that changes will be made to the service and so the assumption should be that changes will occur and that these need to be described*’ [Open round comment]

‘*Need to allow for change in intervention over time as well as local adaptability - these* [questions] *assume new service is fixed in aspic*’ [First scoring round comment]

The importance of describing in some detail the situation in the comparator groups was also emphasised as this could ‘be very different from place to place’.

‘*Components of the new strategy may be part of the “usual care” given in one centre but not in another*.’ [Second scoring round comment]

#### Overlap with other reporting guidelines

A number of comments referred to the large number of existing reporting standards (‘over 25 archived on the EQUATOR network already’ [13]), and a number of respondents raised concerns about overlap with CONSORT [14], STROBE [19], COREQ [32], TIDieR [33] and the ‘danger of “publication guideline fatigue” amongst investigators and journal editors’. It was emphasised that StaRI ‘will need to be clear where it starts and other standards end’ though opinion was divided about whether it was better to ‘cross-reference to’ other guidelines or ‘integrate with’ them.

‘*Perhaps better to defer investigators to these existing guidelines when methods of study (RCT, observational) overlap with existing guidelines*’ [Open round comment]

‘*A review and compilation of the relevant CONSORT statements and extensions should be used to expand the starting list*’ [Open round comment]

## Discussion

### Summary of findings

We found consensus on 35 items as priority items for reporting implementation studies and also identified a number of issues for further discussion. Over-arching themes included balancing the need for a detailed description of complex implementation interventions with the practical demands of writing a concise paper, reflecting the dual aims of reporting the implementation process and effectiveness of the intervention and monitoring fidelity to an intervention whilst enabling modification/adaptation to suit the local context of different centres.

### Strengths and limitations

In line with recognised methodology [23], our study adopted a systematic approach to generate potential standards drawing on both existing literature and expert opinion. A key strength was the breadth of expertise within our international multidisciplinary panel, though we acknowledge that we may not have encompassed all possible perspectives.

We systematically considered all the suggestions from the open round and revised the list of potential standards accordingly in order to reflect the insights provided by the expert panel. Graphical representations of the median scores and the spread of first round scores were fed back to participants to facilitate the process of achieving consensus in the second round. Despite our explicit emphasis during recruitment on the importance of committing to the complete consensus exercise, three participants only contributed to the open round and one respondent withdrew between the two scoring rounds.

### Interpretation

The importance of a detailed description of an intervention has previously been emphasised in the context of RCTs [34-36]. The comments from our expert panel suggest that this is an even more complex challenge for authors of papers reporting implementation studies in which a core intervention may (or possibly should) evolve over time and be adapted to accommodate diversity of sites. Adoption of innovative strategies for describing interventions, such as graphical representation [34], and long-term repositories potentially linked to the trial registration number for additional materials (including, for example, videos, manuals and tools used to assess fidelity in studies) [35,36]. Use of standardised taxonomies [5,36] may be of particular benefit in enabling full descriptions of the implementation process.

A key issue highlighted by our respondents was the large number of existing reporting standards [13] and the increasing potential for overlap between them. Having too many checklists potentially causes confusion as authors and editors are required to select the correct guideline: too few and researchers working with less common methodologies may be forced to ‘shoehorn’ their publication into inappropriate but recognised formats.

Reporting standards are inherently linked with methodology. Methodological considerations will determine standards, but equally, requirements for reporting may influence researchers as they design their studies. In the context of implementation studies, StaRI reporting guidelines not only build on but may also contribute to further revisions of the MRC framework which currently focuses on the development and evaluation of complex interventions [1]. The framework identifies some ‘promising approaches’ to effective dissemination, identifies the need for and offers two examples of implementation studies [37,38], but does not provide detailed guidance. Reporting standards represent expert opinion on key methodological approaches, which may help inform an extension of the MRC framework [11].

Guidelines and comparative effectiveness programmes [39,40] typically prioritise RCTs and rarely recognise the significance of implementation studies in informing health care practice. Poor reporting only exacerbates this problem as potentially important implementation work is either not identified or its importance downgraded.

## Conclusions

The starting point for the StaRI work was the recognition of the poor standard of reporting of implementation work [5,11]. This literature review and e-Delphi exercise represents the first two stages in developing agreed international standards. A workshop is planned in Spring 2015 that will have the remit to discuss the over-arching issues, clarify specific items and develop StaRI reporting standards to fit within the suite of EQUATOR reporting guidelines. If adopted by authors and enforced by editors, the standard should promote consistent reporting of implementation research that can inform health services and health care professionals seeking to implement research findings into routine clinical care.

## Abbreviations

COREQ: Consolidated criteria for reporting qualitative research
EQUATOR: Enhancing the quality and transparency of health research
MRC: Medical Research Council
PRISMA: Preferred reporting items for systematic reviews and meta-analyses
RCT: Randomised controlled trial
STROBE: Strengthening the reporting of observational studies in epidemiology
TIDieR: Template for intervention description and replication
